# Subjective cognitive complaints in patients with stress-related exhaustion disorder: a cross sectional study

**DOI:** 10.1186/s40359-021-00576-9

**Published:** 2021-05-18

**Authors:** Andreas Nelson, Hanna Malmberg Gavelin, Carl-Johan Boraxbekk, Therese Eskilsson, Maria Josefsson, Lisbeth Slunga Järvholm, Anna Stigsdotter Neely

**Affiliations:** 1 Department of Social and Psychological Studies, Karlstad University, Karlstad, Sweden; 2 Department of Anaesthesiology, Central Hospital of Karlstad, Karlstad, Region Värmland Sweden; 3 Academic Unit for Psychiatry of Old Age, University of Melbourne, Melbourne, Australia; 4 Department of Psychology, Umeå University, Umeå, Sweden; 5 Department of Radiation Sciences, Diagnostic Radiology, Umeå University, Umeå, Sweden; 6 Institute of Sports Medicine Copenhagen (ISMC), Copenhagen University Hospital Bispebjerg, Copenhagen, Denmark; 7 Danish Research Centre for Magnetic Resonance, Centre for Functional and Diagnostic Imaging and Research, Copenhagen University Hospital, Amager and Hvidovre, Denmark; 8 Department of Public Health and Clinical Medicine, Section for Sustainable Health, Umeå University, Umeå, Sweden; 9 Department of Community Medicine and Rehabilitation, Physiotherapy, Umeå University, Umeå, Sweden; 10 Centre for Demographic and Ageing Research (CEDAR), Umeå University, Umeå, Sweden; 11 Department of Statistics Umeå, University, Umeå, Sweden; 12 Department of Social Sciences, Technology and Arts, Luleå University of Technology, Luleå, Sweden; 13 Department of Health, Education and Technology, Luleå University of Technology, Luleå, Sweden

**Keywords:** Stress, Burnout, Stress-induced, Exhaustion, Subjective cognitive complaints, Cognition

## Abstract

**Background:**

Stress-related exhaustion is associated with cognitive impairment as measured by both subjective cognitive complaints (SCCs) and objective cognitive test performance. This study aimed to examine how patients diagnosed with exhaustion disorder differ from healthy control participants in regard to levels and type of SCCs, and if SCCs are associated with cognitive test performance and psychological distress.

**Methods:**

We compared a group of patients with stress-related exhaustion disorder (*n* = 103, female = 88) with matched healthy controls (*n* = 58, female = 47) cross-sectionally, concerning the type and magnitude of self-reported SCCs. We furthermore explored the association between SCCs and cognitive test performance as well as with self-reported depression, anxiety and burnout levels, in the patient and the control group, respectively.

**Results:**

Patients reported considerably more cognitive failures and were more likely than controls to express memory failures in situations providing few external cues and reminders in the environment. In both groups, SCCs were associated with demographic and psychological factors, and not with cognitive test performance.

**Conclusion:**

Our findings underline the high burden of cognitive problems experienced by patients with exhaustion disorder, particularly in executively demanding tasks without external cognitive support. From a clinical perspective, SCCs and objective cognitive test performance may measure different aspects of cognitive functioning, and external cognitive aids could be of value in stress rehabilitation.

**Trial registration:**

Participants were recruited as part of the Rehabilitation for Improved Cognition (RECO) study (ClinicalTrials.gov: NCT03073772). Date of registration: 8 March 2017

**Supplementary Information:**

The online version contains supplementary material available at 10.1186/s40359-021-00576-9.

## Introduction

Stress-related illness is one of the main reasons for sick-leave in Europe today [1, 2]. A well-known construct describing the sequalae of long-term stress is burnout, characterized by mental exhaustion, increased mental distance to one’s job and reduced personal accomplishment [3]. Since burnout refers to an occupational phenomenon and not to a clinical condition [4], the Swedish version of the International Classification of Diseases (ICD-10-SE) has introduced exhaustion disorder (ED; code F43.8) as a clinical equivalent of the same stress reaction, while including also non-work stressors. Consequently, patients with ED score high levels of burnout [5].

The diagnostic criteria for ED include problems with memory or concentration and stress-related exhaustion has been associated with elevated levels of subjective cognitive complaints (SCCs) [6–9], as well as with suboptimal cognitive performance in executive functions, attention, working memory and processing speed [5, 10]. Yet, the specific nature of the cognitive complaints reported by ED patients is unclear, as is the relationship between SCCs, cognitive performance and psychological distress. Firstly, the few studies that have examined the link between SCCs and cognitive performance in this group have shown no or low correlations between these measures [9, 11–14]. Notably, a weak relationship between SCCs and cognitive performance is not exclusive for ED and has been highlighted in several other clinical groups, for example in chronic fatigue syndrome, mood disorders, and in healthy and pathological aging [15–18]. Secondly, in several populations, SCCs have been found highly correlated with psychological and demographic factors such as anxiety, depression, age and sex and it has been suggested that SCCs reflect these phenomena rather than objective cognitive dysfunction [19–21]. While this question is less studied in the context of stress-related exhaustion, SCCs have been associated with increased levels of depression in this group [9, 13, 14]. Thirdly, previous studies on stress-related exhaustion have operationalized SCCs using single items [11, 12] or total scores on questionnaires targeting everyday memory or cognitive functioning [7–9, 22, 23]. Although these measures indicate a general experience of cognitive deficit, they do not provide information on the specific type of complaints experienced by ED patients. To this end, we included the Prospective and Retrospective Memory Questionnaire (PRMQ), a validated instrument allowing comparisons between types of memory failures along three dimensions: (a) prospective vs retrospective memory, (b) self-cued vs environmental cued memory and (c) short-term vs long-term memory, thus providing a more comprehensive understanding of SCCs in patients with ED [24, 25].

The main objective of this study was to examine SCCs in a sample of patients diagnosed with ED compared to a healthy control group. Specifically, we aimed to investigate:

1. The magnitude of SCCs reported by patients diagnosed with ED, compared to healthy control participants.
2. If patients with ED differ from healthy control participants in type of SCCs.
3. If SCCs are associated with cognitive test performance and psychological distress in patients with ED and in a healthy control group, respectively.

## Methods

### Participants

The 103 patients included in this study were part of the RECO study, a randomized clinical trial conducted at the Stress Rehabilitation Clinic at the University Hospital in Umeå, Sweden (ClinicalTrials.gov: NCT03073772). Results as well as detailed methodological descriptions have previously been presented elsewhere [22, 23, 26, 27]. Briefly, this three-armed trial examined the effects of cognitive and aerobic training as add-on interventions of a 24-week multimodal stress rehabilitation (MMR) programme. This study analysed data from the pretest assessments which took place after twelve weeks of MMR and before randomization to the experimental arms (see Additional file 1: Fig S1 for the flow of patients and attrition). A 132 patients partook in the pretest assessment, where 29 patients had missing data on SCCs and/or cognitive test data and were not included in this study, making the total sample 103 patients. As shown in Additional file 1: Table S1, dropout analysis revealed no differences between the analysed sample of patients (*n* = 103) and those excluded due to missing cognitive data (*n* = 29) in regard to age, sex, education, burnout levels and verbal ability. At start of the trial, 161 patients agreed to participate in the study and filled out some questionnaires as an initial baseline assessment. A dropout analysis comparing the patients remaining in the study (*n* = 132) with the patients who dropped out before pretest (*n* = 29) yielded no differences in age, sex, education, or burnout levels (Additional file 1: Table S2).

Patients were screened for eligibility and recruited from the Stress rehabilitation clinic and the Social insurance agency in Umeå, Sweden from April 2010 until June 2013. Inclusion criteria for the patients were (1) exhaustion disorder diagnosis, confirmed by a physician and a psychologist; (2) 18–60 years old; (3) currently employed; (4) considered by a physician and a psychologist to be suitable for group-based stress rehabilitation; (5) no known abuse of alcohol or drugs; (6) not in need of more urgent treatment; and (7) not participating in other interventional study. Patients with relevant diagnoses in addition to ED (e.g., neurological or chronic psychiatric diagnoses) that required special care and treatment adjustments were not considered suitable for the standardized group-based stress rehabilitation, and therefore not included in this study.

All patients fulfilled the criteria for Exhaustion disorder (ED; see Table 1) based on diagnostic criteria established by the Swedish National board of Health and Welfare in 2005 and were assigned the F43.8A code of the International Classification of Diseases and Related Health problems (ICD-10) [28]. All patients were on partial or fulltime sick-leave when recruited to the study.

**Table 1:** Diagnostic criteria for exhaustion disorder, ICD-10-SE code F43.8A

A. Physical and mental symptoms of exhaustion with a minimum of 2 weeks duration. The symptoms have developed in response to one or more identifiable stressors, present for at least 6 months
B. Markedly reduced mental energy, which is manifested by reduced initiative, lack of endurance or increase of time needed for recovery after mental efforts
C. At least four of the following symptoms have been present most of the day, nearly every day, during the same 2-week period:
1. Persistent complaints of impaired memory or concentration
2. Markedly reduced capacity to tolerate demands or to work under time pressure
3. Emotionally instability or irritability
4. Disturbed sleep
5. Persistent complaints of physical weakness or fatigue
6. Physical symptoms such as muscular pain, chest pain, palpitations, gastrointestinal problems, vertigo, or increased sensitivity to sounds
D. The symptoms cause clinically significant distress or impairment in occupational, social or other important areas of functioning
E. The symptoms are not due to the direct physiological effects of a substance (e.g., drug abuse, medication) or a general medical condition (e.g., hypothyroidism, diabetes, infectious disease)
F. If criteria for major depressive disorder, dysthymic disorder or generalized anxiety disorder are met, ED is set as co-morbid condition

All criteria signified by capital letters need to be fulfilled for the diagnosis

The control group was recruited in the spring of 2016 through advertisement in a local newspaper inviting healthy participants aged 35–55 to partake in a study on the relationship between every-day stress and cognition. A 109 individuals responded and the first 60 who matched the patient group on age and sex were invited to participate. Initial telephone screening was done to exclude those with self-reported history of medical and/or psychological conditions known to affect cognition, such as cardiovascular, neurological and psychiatric diseases, and stress-related exhaustion. Four control participants were excluded due to prior psychiatric or neurological complications.

The study was conducted in accordance with the Declaration of Helsinki and approved by the Umeå regional ethical review board (Dnr 2010-53-31, 2015-475-32M). Written informed consent was provided by all participants before entering. All participants received financial compensation of 600 SEK for participation.

## Measures and procedures

### Subjective cognitive complaints

SCCs were measured using Swedish translations of two validated questionnaires: the PRMQ and the Cognitive failure questionnaire (CFQ) [24, 29]. The PRMQ consists of 16 items describing everyday memory failures. Answers are given on a Likert scale ranging from never (*1*) to very often (*5*). The results were analysed as the total score, with a possible range between 16 and 80, expressing a general memory factor, as well as six subscales representing three contrasting pair of categories: prospective vs retrospective, short-term vs long-term and self-cued vs environmentally cued memory failures. Prospective memory refers to remembering to perform an intended action (e.g., to deliver a message to a person) whereas retrospective memory refers to remembering previously learnt information (e.g., if the message was delivered in the past). Short-term and long-term memory refer to remembering information or events that was learnt within a few minutes’ time or longer, respectively. Self-cued memory failures may be exemplified as one forgetting to deliver a message to a person when not in the presence of that person, as opposed to being in the presence of that same person (environmentally cued). Each subscale comprises eight items and all items are consequently categorized in all three dimensions. For example, the item “Do you decide to do something in a few minutes’ time and then forget to do it?” is classified as a prospective, short-term and self-cued memory failure, whereas “Do you fail to recognize a place you have visited before?” is categorized as being retrospective, long-term and environmentally cued. Cronbach’s alphas for the respective scales were: 0.94 (total score) 0.91 (prospective), 0.87 (retrospective), 0.89 (short-term), 0.88 (long-term), 0.90 (self-cued) and 0.87 (environmentally cued). A detailed account of the scale structure and a list of the specific items are found in Crawford et al. [24]. Psychometric properties of the Swedish adaptation are described in Rönnlund et al. [25]. The CFQ targets cognitive failures in daily life and consists of 25 items presented in a five-point Likert scale format ranging from never (*1*) to very often (*5*). Results are presented as a total score with a possible range between 0 and 100 (Cronbach’s alpha: 0.94). The CFQ was included in order to derive a more reliable and comprehensive measure of SCCs by creating a composite score comprising both the PRMQ and the CFQ.

### Cognitive performance

The cognitive test battery consisted of eleven untrained tests measuring executive functions, working memory, episodic memory, perceptual speed and reasoning ability, and has previously been described in detail [27]. Briefly, the test sessions were conducted individually, most often during office hours and lasted two to three hours. The patients were tested between April 2010 and June 2013, and the control group between January and March 2016. Five clinical psychologists/trained research assistants administered the cognitive tests to the patients and two trained research assistants tested the control participants. The tests were administered in the following order: Digit symbol, Letter memory running span, Digit span forwards, Digit span backwards, Colour-word interference test, Trail making test, *n*-back, Letter-number sequencing, Raven’s matrices, Recall of concrete nouns, and the Visuospatial span task (which could not be analysed due to technical reasons).

#### Executive functions

Inhibition ability was measured using the Colour-word interference test (also called the Stroop test) from the Delis-Kaplan executive function system (D-KEFS) test battery [30]. The outcome measure was inhibition cost (time in sec to complete incongruent reading compared to reading of words denoting colours). Shifting ability was measured using the Trail making test, also from D-KEFS. The outcome was defined as shift cost (the time in seconds used to complete the shifting condition compared to the time used to complete the number-sequencing condition). Updating was measured as performance on the *n*-back task and on the Letter memory running span task [31]. In *n*-back, lists of single digits (1–9) were presented to the participants whom were instructed to report whether a digit matched the one presented n steps back. The outcome measure was 3-back accuracy (hits–false alarms). In Letter memory running span, participants were presented with lists of single letters (A–D) on a computer screen. Following the presentation, participants were instructed to recall the last four presented letters in correct order. Outcome was defined as the total number of correctly recalled four-letter sequences across ten presentations.

#### Working memory

Working memory was assessed using Digit span forward and Digit span backward from the Wechsler adult intelligence scale (WAIS)-R [32] and Letter-number sequencing from WAIS-III [33]. The outcome measure was the total number of correctly recalled sequences in each condition.

#### Episodic memory

Episodic memory was operationalized as ability to recall a list of 18 nouns, administered and interpreted according to Buschke’s procedure of selective reminding [34]. Outcome measure was the total number of recalled nouns across four presentations.

#### Perceptual speed

Perceptual speed was tested using Digit Symbol from WAIS-R [32]. The total number of correctly drawn symbols in 90 s was used as the outcome measure.

#### Reasoning ability

Raven’s advanced progressive matrices were used as a measure of reasoning ability [35]. The test consists of 36 pattern matrices and was split into two parts using odd and even items. Outcome measure was the total number of correctly solved matrices in 10 min.

### Psychological distress

#### Burnout

Level of burnout was measured using the Shirom-Melamed burnout questionnaire (SMBQ) [36, 37]. This validated instrument consists of 22 items rated on a 7-point Likert scale (1 = *almost never*, 7 = *almost always*) and generates five outcome measures: a total mean score of all items (Cronbach’s alpha: 0.98) and four subscales. An emotional exhaustion/ physical fatigue scale (hereafter referred to as Exhaustion) comprises 8 items (e.g., “I feel physically exhausted”, “My batteries are dead”) (Cronbach’s alpha: 0.86), a tension scale of 4 items (e.g., “I feel intense inner tension”) (Cronbach’s alpha: 0.90), a listlessness scale of 4 items (e.g., “I feel sleepy”) (Cronbach’s alpha: 0.93) and a cognitive weariness scale of 6 items (e.g., “I feel I am disorganized lately”) (Cronbach’s alpha: 0.96). Higher scores indicate more symptoms.

#### Depression and anxiety

Depression and anxiety were assessed by the Hospital anxiety and depression scale (HADS) [38]. This questionnaire comprises 14 items rated on a four-point Likert scale (0–3), targeting either anxiety or depression. The total score on each scale (range 0–21) was used as the outcome measure, with a higher score indicating more symptoms. Cronbach’s alpha was 0.81 for the depression subscale and 0.78 for the anxiety subscale.

### Statistical analyses

All statistical analyses were performed using IBM SPSS Statistics (version 25). To make variables comparable, standardized z-scores ($z=\frac{x-\mu }{\sigma }$) were calculated using the control group as a reference, i.e., their mean ($\mu$) and standard deviation $\left(\sigma \right)$. For demographic characteristics, differences between the groups were analysed using independent samples t-test for age and Pearson’s chi-squared tests for education and sex. In order to investigate mean differences, independent samples t-tests were used for the psychological distress variables and one-way analyses of variance (ANOVAs) for the cognitive test performance and SCCs variables, respectively. Effect sizes (Glass’s Δ) were computed as the mean difference between the patient group and control group, divided by the standard deviation of the control group.

Four composite scores were calculated to analyse differences in performance between groups: (a) a global cognitive score comprising all ten cognitive tests; (b) a working memory domain score comprising Digit span forward, Digit span backward and Letter-number sequencing; (c) an executive functions domain score comprising the Colour-word interference test, the Trail making test, the *n*-back task and the Letter memory running span task; (d) a summed SCCs score computed as the mean value of the standardized PRMQ and CFQ total scores. The inhibition cost of the Colour-word interference test and the shift cost of the Trail making test were reversed so that higher scores indicated better performance. The episodic memory, perceptual speed and reasoning ability domain scores comprised singular cognitive tests, i.e., Recall of concrete nouns, Digit symbol and Raven’s progressive matrices, respectively. All domain and composite scores were based on z-scores. An imputation procedure was used to replace missing responses on single items, before calculating the composite scores, using an expectation–maximization with bootstrapping method, as implemented in the Amelia II software package [39, 40]. For the cognitive tests, this was done for three participants who were missing single test results. For the measures of SCCs, three items (0.23%) were imputed on the PRMQ. Participants missing responses to more than two items on the PRMQ were excluded from analysis. We included age, sex and the single items for each cognitive test separately in the imputation. To analyse differences in the type of cognitive complaints reported by patients and controls, a 2 (Group: patients, controls) X 2 (Type of cognitive complaint) mixed ANOVA was performed respectively for each type of complaint (prospective vs retrospective memory failures, self-cued vs environmentally cued memory failures, short-term vs long-term memory failures). The within group factor was Type of cognitive complaint. Three-stage hierarchical multiple regression analysis was conducted in the patient group and control group, respectively, with the SCCs composite being the dependent variable in both models. Age, sex and education level were entered at stage one of the regressions in order to control for demographic variables. Cognitive test performance (the global cognitive composite) was entered at stage two and the psychological variables (exhaustion, depression and anxiety) at stage three. The exhaustion scale of the SMBQ was chosen because it, unlike the total score, does not comprise items describing cognitive weariness, thereby enabling meaningful statistical analysis of the relation between burnout and SCCs. All variables were *z*-transformed with the exception of sex and education level, which were dummy-coded and entered as categorical variables. Education level was dummy coded as 1 = University and 0 = Other. Lastly, in order to examine if any specific type of complaint was correlated to any of the cognitive domains, partial correlation analyses were performed for patients and controls respectively, controlling for age, sex and education level.

## Results

### Demographic and psychological variables

Demographic and clinical characteristics of the patient group and control group are presented in Table 2. The groups were similar in age, sex and education level. Patients showed significantly more self-reported symptoms of burnout, depression and anxiety. All effect sizes were large (Glass’s Δ > 2).

**Table 2:** Demographic and clinical characteristics

Variable	Patients (n = 103)	Control group (n = 58)	*df*	*χ *^*2*^	*t*	*p*	*Δ*
Female^b^	88 (85.44%)	47 (81.03%)	1	0.53		.47	
Age^a c^	43.28 (8.75)	43.74 (6.59)	159		0.35	.73	
Range	22–60	35–56					
Education^b^			2	1.06		.59	
Elementary school	6 (5.83%)	2 (3.45%)	159				
High school	32 (31.07%)	22 (37.93%)	159				
University	65 (63.11%)	34 (58.62%)	159				
SMBQ^a c^							
Total score	4.87 (0.96)	2.47 (0.91)	159		− 15.53	*.00*	2.16
Exhaustion	4.71 (1.15)	2.40 (0.92)	159		− 13.21	*.00*	2.10
Listlessness	5.00 (1.15)	2.67 (1.20)	159		− 12.17	*.00*	2.78
Tension	4.86 (1.10)	2.59 (1.28)	159		−11.80	*.00*	2.83
Cognitive weariness	5.01 (1.17)	2.34 (1.18)			− 13.83	*.00*	3.02
HADS^a c^							
Depression	6.87 (3.46)	2.16 (1.84)	159		− 9.63	*.00*	2.56
Anxiety	9.72 (3.56)	4.26 (3.05)	159		− 9.82	*.00*	1.79

Significant group differences are italicized

^a^Mean (SD). ^b^Based on Pearson’s Chi-square test. ^c^Based on Independent Samples T-test

### Group differences in subjective cognitive complaints and test performance

**Fig. 1:**
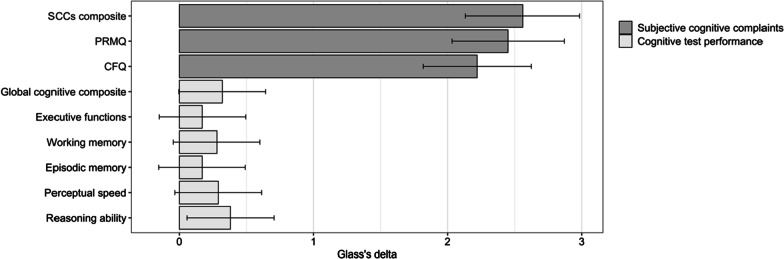
Effect sizes of differences between patients and controls in SCCs and cognitive test performance. Note*:* Bars indicate group differences in Glass’s Δ. Error bars indicate confidence intervals (95%). *p < .05. **p < .01

Table 3 shows results of the analysis of group differences in SCCs. The one-way ANOVA revealed that the patients reported significantly more SCCs than did the controls. This held true regarding both the PRMQ and the CFQ. As shown in Fig. 1, the effect sizes of the group differences were large for all measures of SCCs (Glass’s Δ > 2). Table 4 presents the results of the ANOVA for all cognitive tests. Compared to controls, patients performed significantly worse on the *n*-back task, the Letter-number sequencing task and Raven’s matrices. Patients performed better than controls on the Letter memory running span task. No other significant group differences were found although the global cognitive composite was close to statistical significance (0.051), indicating worse overall performance by the patients. The effect sizes of group differences in the respective cognitive domains ranged between 0.17 and 0.38 (Fig. 1).

**Table 3:** Group differences in subjective cognitive complaints

Variable	Patients		Control group		*F (df)*	*p*
	*M*	*SD*	*M*	*SD*		
SCCs composite^a^	2.56	1.40	0.00	1.00	150.45 (1,159)	*.00*
PRMQ, total score^b^	49.37	10.76	32.48	6.89	115.98 (1,159)	*.00*
CFQ, total score^c^	55.50	11.99	32.19	10.50	153.09 (1,159)	*.00*

Significant group differences are italicized

^a^Z-scores. ^b^Range 16–80. ^c^Range 0–100

**Table 4:** Group differences in cognitive test performance

Variable	Patients		Control group		*F (df)*	*p*
	*M*	*SD*	*M*	*SD*		
Global cognitive composite	− 0.32	0.99	0.00	1.00	3.85 (1,159)	.05
Executive functions	− 0.17	1.10	0.00	1.00	0.98 (1,159)	.32
Letter memory running span	2.42	1.68	1.71	1.49	7.18 (1,159)	*.01*
*n*-back	21.18	7.32	23.93	6.63	5.58 (1,159)	*.02*
Stroop inhibition cost^a^	30.21	11.75	26.88	8.63	3.58 (1,159)	.06
TMT shift cost^a^	48.93	25.00	45.14	25.52	0.84 (1,159)	.36
Working memory	− 0.28	0.98	0.00	1.00	2.93 (1,159)	.09
Digit span forwards	6.99	2.06	6.64	1.74	1.21 (1,159)	.27
Digit span backwards	6.45	2.09	7.00	2.06	2.62 (1,159)	.11
Letter-number sequencing	9.81	2.56	11.69	2.92	18.20 (1,159)	*.00*
Episodic memory	− 0.17	1.04	0.00	1.00	1.00 (1,159)	.32
Recall of concrete nouns	51.08	9.71	52.66	9.37	1.00 (1,159)	.32
Perceptual speed	− 0.29	1.01	0.00	1.00	3.08 (1,159)	.08
Digit symbol	52.85	10.23	55.79	10.15	3.08 (1,159)	.08
Reasoning ability	− 0.38	0.95	0.00	1.00	5.74 (1,159)	*.02*
Raven’s matrices	6.92	2.86	8.07	3.01	5.74 (1,159)	*.02*

Left aligned variables show composited domains as z scores. Indented variables show the individual tests within the composite. Significant group differences are italicized

^a^Higher scores indicate worse performance

### Type of subjective cognitive complaint

#### Prospective vs retrospective memory failures

**Fig. 2:**
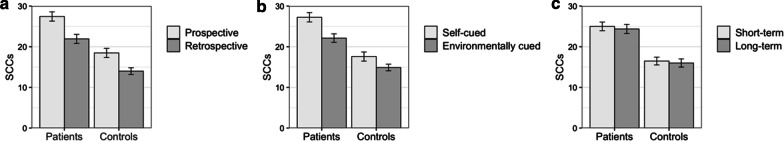
Mean of reported memory failures for patient and control participants. Note*:* Error bars indicate confidence intervals (95%)

The 2 (Group: patient, control) × 2 (Type of cognitive complaint: retrospective, prospective memory) mixed ANOVA revealed a significant main effect of group, *F* (1, 159) = 115.98, *p* = 0.00, *η*_*p*_^*2*^ = 0.42, showing that patients expressed more memory complaints than did controls. It also revealed a significant main effect of Type of cognitive complaint, *F* (1,159) = 261.77, *p* = 0.00, *η*_*p*_^*2*^ = 0.62, showing more prospective than retrospective memory failures. The interaction effect, indicating a tendency for patients to report disproportionally more prospective than retrospective memory problems, fell short of significance, *F* (1, 159) = 2.74, *p* = 0.10. See Fig. 2a for the results.

#### Self-cued vs environmentally cued memory failures

The 2 (Group: patient, control) × 2 (Type of cognitive complaint: self-cued, environmentally cued) mixed ANOVA revealed a significant main effect of group, *F* (1, 159) = 115.98, *p* = 0.00, *η*_*p*_^*2*^ = 0.42, showing that patients expressed more memory failures than did controls. It also revealed a significant main effect of Type of cognitive complaint, *F* (1,159) = 231.19, *p* = 0.00, *η*_*p*_^*2*^ = 0.59, showing more self-cued than environmentally cued memory failures. More importantly, the interaction effect was significant, *F* (1,159) = 22.35, *p* = 0.00, *η*_*p*_^*2*^ = 0.12, reflecting that the patients reported disproportionally more self-cued than environmentally-cued problems (*p* < 0.05) compared to controls (p < 0.05). Figure 2b shows the results.

#### Short term vs long term memory failures

The 2 (Group: patient, control) × 2 (Type of cognitive complaint: short-term, long-term memory) mixed ANOVA revealed a significant main effect of group, *F* (1, 159) = 115.98, *p* = 0.00, *η*_*p*_^*2*^ = 0.42, showing that patients expressed more memory failures than controls. A significant effect of Type of cognitive complaint was found (*F* (1,159) = 5.012, *p* = 0.03, *η*_*p*_^*2*^ = 0.03, displaying more short-term than long-term memory failures. No significant interaction effect between group and Type of complaint was found, *F* (1, 159) = 0.09, *p* = 0.7. Figure 2c displays the results.

## Associations between SCCs, cognitive test performance and psychological distress

### Patients

In the patient sample, the hierarchical multiple regression revealed that the combined demographic variables at stage one significantly contributed to the regression model (*F* (3,99) = 2.81, *p* = 0.04) and that the cognitive test performance variable entered at stage two did not. The addition of the psychological distress variables at stage three explained an additional 9.5% to the model, showing that higher levels of psychological distress were associated with more SCCs, and contributed significantly to the model (*F* (3,95) = 3.64, *p* = 0.02). The individual variable that contributed most to the model was age. Lower age was associated with more SCCs. Together, the independent variables in the final regression model accounted for 18% of the variance in SCCs.

### Control group

Similarly, the hierarchical multiple regression on the control participants showed that the demographic variables at stage one significantly contributed to the regression model (*F* (3,54) = 2.91, *p* = 0.04) and that the cognitive test performance variable entered at stage two did not. At stage three, the addition of the psychological variables explained an additional 25.8% to the model and this change in R2 was significant, *F* (3,50) = 7.24, *p* = 0.00. The individual variable that contributed most to the model was sex. Female sex was associated with more SCCs. Together, all independent variables in the regression model accounted for 41% of the variance in SCCs. Results from the hierarchical regression models are presented in Table 5. Pearson correlations of the variables in the regression analyses are presented in Additional file 1: Table S3.

**Table 5:** Summary of hierarchical regression analyses showing associations with subjective cognitive complaints

Variable	Patients						Control group					
	β	*SE*	*p*	*R*	*R*^*2*^	*ΔR*^*2*^	β	*SE*	*p*	*R*	*R*^*2*^	*ΔR*^*2*^
Step 1			*.04*	.28	.08	.08			*.04*	.37	.14	.14
Age	− .25	0.10	*.01*				− .05	0.13	.70			
Sex												
Female	.06	0.38	.54				.38	0.33	*.01*			
Education level												
University	.13	0.28	.19				.02	0.26	.88			
Step 2			.65	.28	.08	.00			.46	.39	.15	.01
Global cognitive composite	.05	0.15	.65				.10	0.14	.46			
Step 3			*.02*	.42	.18	.10			*.00*	.64	.41	.26
Exhaustion	.12	0.14	.36				.16	0.14	.26			
Depression	.20	0.09	.11				.31	0.17	.07			
Anxiety	.04	0.14	.75				.15	0.17	.39			

Reported beta coefficients are from the step in which the variables were first entered. Significant results are italicized

Additional file 1: Table S4 shows partial correlations between all scales of the PRMQ and the CFQ and all tested cognitive domains, controlling for age, sex and education level. No significant correlations were found between any measure of SCCs and any cognitive domain, in the patient sample and control group, respectively.

## Discussion

The aims of this study were to investigate the level and type of SCCs in patients with ED compared to healthy control participants, and if SCCs were associated with cognitive test performance and psychological distress in patients with ED and in a healthy control group, respectively.

Patients expressed more SCCs than controls on all scales of the PRMQ and on the CFQ and the interaction analysis revealed that patients reported more self-cued than environmentally cued memory failures. Self-cued memory tasks are considered more dependent on cognitive control abilities, and the results may thus indicate that patients with ED are more prone to report memory failures in tasks dependent on executive functioning [41]. This finding is in accord with previous research showing worse performance by patients with ED on prospective memory tasks constructed to exclude [6] or manipulate the degree of [8] external cues in order to increase the demand of executive cognitive control.

A subjective experience of failure in executively demanding tasks harmonize with the cognitive profile suggested by the existing literature on stress-related exhaustion [10] and also with the cognitive test results of this study, as we found that patients showed impaired cognitive performance on individual tasks assessing executive function and working memory, as well as reasoning ability, which is associated with executive functioning [42]. In contrast, patients performed better than controls on the Letter memory running span task, also tapping executive functioning. It should be noted, however, that the patients involved in this study were admitted to 12 weeks of rehabilitation before the cognitive assessment, and that diverse findings concerning cognitive test performance in stress-related exhaustion are not unique to this study. Previous research has commonly, but not always, found stress-related exhaustion to be associated with executive impairment, and the specific tasks and functions shown affected have varied between studies [10]. While there are several possible explanations for this heterogeneity, it has been suggested that worse test performance by patients with ED may appear following more prolonged test procedures [43]. It is therefore notable that Letter memory running span was one of the first tests to be administered during the two-hour test session, whereas the three tasks showing significantly worse performance by patients were among the last, perhaps indicating an influence of fatigue on the results.

Although the results of both subjective and some of the objective cognitive measures suggest impairment in ED patients, the magnitude of the group differences is notably dissimilar when comparing these different measures. As displayed in Fig. 1, effect sizes were large for SCCs, contrasting small effect sizes for cognitive test performance. Such pattern, with group differences being greater in subjective than in objective cognitive measures, has previously been shown in studies on stress-related exhaustion [8, 9, 44] and may suggest a need for more ecologically valid measures to capture the everyday cognitive difficulties experienced by patients with ED.

The regression analyses revealed that SCCs were associated with psychological distress, supporting the results by Österberg et al. [9, 13, 14] showing an association between SCCs and depression in patients with stress-related exhaustion. However, not only depression drove this effect in our data. The psychological distress variables entered in the regression models were highly intercorrelated and when entered individually, all were significantly associated with SCCs (data not shown). Thus, the present study cannot elucidate which singular psychological distress aspect that is most strongly linked to SCCs, possibly reflecting that a general level of distress, rather than any specific effect of depression, anxiety or burnout symptoms, is driving this effect.

In both the patient sample and the control group, all scales of the PRMQ and the CFQ were unrelated to all cognitive domains when controlling for age, sex and education level. This aligns with the results of previous studies that have found no or low correlations between test performance and SCCs in patients with stress-related exhaustion [9, 11–14]. Such weak relationship between subjectively and objectively measured cognition has been shown in several populations, for example aging [19], fibromyalgia [15] and chronic fatigue syndrome [18], with explanations varying among authors, often involving specific traits of the studied clinical population, such as depression, poor cognitive appraisal ability, memory perfectionism, overinterpretation of everyday distractibility or enhanced self-monitoring [e.g., 9, 14, 45, 46].

While our results confirm that SCCs are indeed associated with depression and other measures of psychological distress, they moreover reveal that cognitive test performance and SCCs are unrelated in the control group. This finding is consistent with the existing literature on healthy populations, which have failed to show a consistent link between self-reported cognitive failures and objective cognitive assessment [20, 47] and hence contradict the notion that the weak relationship between subjective and objective cognitive measures is due to any exclusive feature of the clinical population**.** In this regard, it should be noted that a study by Stenfors and colleagues [47] examining SCCs and executive functioning in a healthy working population-based sample found results in line with ours, suggesting that the pattern of results are robust despite differences in recruitment strategy (i.e., clinical vs population-based samples).

One possible explanation for the weak association between SCCs and cognitive test performance is that they are indicators of partly distinct cognitive phenomena [48]. Performance based instruments, such as the tests used in this study, normally target optimal performance under highly structured conditions where the goals and outcomes of the task are pre-decided and integral to the standardized format. Consequently, tests of optimal performance provide information about the efficiency of cognitive information processing in achieving the prerequisite goal. They do not, however, measure how to decide and pursue goals in everyday situations. In contrast, rating-based measures provide far fewer external cues on how to interpret the task and its outcome, and may tap a reflective cognitive level directly concerned with the actual real-life goals and beliefs of the individual [48].

Notably, a key difference between rating- and performance-based measures is the degree to which external structure is provided. This is interesting considering the result that ED patients reported more everyday memory failures in situations providing fewer external cues, and indicates that compensation through external support may have important clinical implications. We have previously suggested that patients with ED may be able to compensate for cognitive deficits through increased mental effort, as reflected by recruitment of additional neuronal resources during cognitive task performance [26], potentially adding to the state of exhaustion. If ED patients were to compensate for cognitive deficits by easing the demands of everyday tasks through external support, instead of merely trying harder, this may help aid not only cognitive functioning but also the core symptom of mental exhaustion. Therapies that include cognitive support strategies, such as attention recruitment and cue-based reminders, are common in care of neurological conditions and have shown promising results in treatment of depression [49, 50]. The results from this study suggest that such interventions may have clinical value also in rehabilitation of ED.

Furthermore, female sex was significantly associated with more SCCs in the control group, which is in agreement with previous research on healthy populations [20, 51]. No such sex effect was revealed in the patient sample, indicating that male and female ED patients expressed similarly high levels of SCCs. These results need to be interpreted with caution considering the small number of male participants in our study. It is worth noting, however, that the findings accord with Österberg et al. [9] who found larger differences in SCCs between patients with stress-related exhaustion and a reference group among men compared to among women. Also, younger age was associated with more SCCs in the patient sample but not in the control group. Perhaps counterintuitive, the latter result harmonizes with previous research failing to show robust evidence for age-associated increase in SCCs in healthy populations [20, 52]. Possibly, younger ED patients may experience more cognitive failures due to an environment involving additional stressors in regard to both work and family life.

Some limitations of this study need to be addressed. Firstly, the cross-sectional design disallows drawing causal inferences and analyses of change over time. This is noteworthy considering that subjective and objective cognitive measures have been found to be more strongly related when assessing longitudinal change [53]. As alluded to above, the patients in this study received 12 weeks of multimodal stress rehabilitation before the assessment of subjective and objective cognition which needs to be taken into consideration when interpreting our results. It is conceivable that slight improvements in cognition and psychological health may have occurred during this time, leading to an underestimation of the differences between groups. Moreover, the time course of improvement may be different for subjective and objective cognition [54], underlining the importance of longitudinal investigations of the interrelations between SCCs and cognitive performance. Furthermore, while the groups were matched and similar in several important aspects, some differences were not controlled for. For instance, the groups were recruited at different time points and by different recruitment strategies. Lastly, while an extensive test battery and several measures of SCCs provide detailed information on cognition in this group, it also increases the risk of chance findings, as we chose not to correct for multiple comparisons due to the increased risk of making a type-II error.

Strengths of this study include the relatively large sample size and the broad cognitive test battery as well as the inclusion of a matched group of healthy controls. The patient sample was also well-defined and recruited from a clinical setting. A further strength was the incorporation of two validated self-report measures of everyday cognitive failures: the PRMQ, allowing a detailed analysis of everyday memory failures along three dimensions, and the CFQ, confirming the magnitude of SCCs in this sample.

## Conclusions

To conclude, this study has provided new insights into the nature of SCCs in patients with ED by showing that ED patients reported more cognitive failures in daily life, most notably in executively demanding tasks lacking external retrieval cues, but performed only slightly worse on cognitive tests when compared to a healthy control group. Also, SCCs were more strongly associated with psychological distress than with cognitive performance in both patients and controls. These findings have implications for clinical practice, as the diagnostic criteria for ED include problems with memory or concentration (ICD-10-SE), commonly assessed through self-report. SCCs and cognitive test performance may provide different types of information on cognitive performance in these patients (i.e., everyday vs. optimal cognitive function). Traditional cognitive tests might therefore fail to detect the everyday cognitive difficulties experienced by ED patients, demonstrating a need for new tools of cognitive assessment, capable of weighing the effects of longer test procedures and subsequent fatigue as well as the aid of external support strategies. Likewise, SCCs may be a suboptimal measure of cognitive performance and we caution against using SCCs as a proxy for cognitive test results. Instead, SCCs provide a window into everyday life functioning and psychological distress tendencies. Future research should further look into why SCCs have a weak relation to objective cognitive performance by using longitudinal designs and by investigating associations with neural correlates.

## Supplementary Information

**Additional file 1.** **Table S1.** Pretest comparisons between the analysed sample of patients (n = 103) and the patients excluded due to missing cognitive data (n = 29). **Table S2.** Baseline comparisons between the patients remaining in the study (n = 132) and the patients who dropped out before pretest (n = 29). **Table S3.** Correlations between the variables included in the regression analyses. **Table S4.** Partial correlations between subjective cognitive complaints and objective cognitive test results, controlling for age, sex and education level. **Figure S1.** Flow and attrition of patients participating in the RECO trial.

## Data Availability

The datasets generated and/or analysed during the current study are not publicly available due to Swedish law (the Swedish Ethical Review Act: 2003:460), but are available from the authors on reasonable request. For such requests, please contact Anna Stigsdotter Neely or Lisbeth Slunga Järvholm.

## Abbreviations

ANOVA: Analysis of variance
CFQ: Cognitive failure questionnaire
D-KEFS: Delis-Kaplan executive function system
ED: Exhaustion disorder
ICD: International classification of diseases
ICD-10-SE: Swedish version of the International Classification of Diseases
HADS: Hospital anxiety and depression scale
MMR: Multimodal stress rehabilitation
PRMQ: Prospective and retrospective memory questionnaire
RECO: Rehabilitation for improved cognition
SCCs: Subjective cognitive complaints
SMBQ: Shirom-Melamed burnout questionnaire
WAIS: Wechsler adult intelligence scale
