# Supplementary material for: Subjective cognitive complaints in patients with stress-related exhaustion disorder: a cross sectional study
Source: BMC Psychol. 2021 May 18;9:84. doi: 10.1186/s40359-021-00576-9 (PMC8132387; doi:10.1186/s40359-021-00576-9)
Supplement: Supplementary file 1 — Additional file 1. Table S1. Pretest comparisons between the analysed sample of patients (n = 103) and the patients excluded due to missing cognitive data (n = 29). Table S2. Baseline comparisons between the patients remaining in the study (n = 132) and the patients who dropped out before pretest (n = 29). Table S3. Correlations between the variables included in the regression analyses. Table S4. Partial correlations between subjective cognitive complaints and objective cognitive test results, controlling for age, sex and education level. Figure S1. Flow and attrition of patients participating in the RECO trial. [file 40359_2021_576_MOESM1_ESM.docx]

**Subjective cognitive complaints in patients with stress-related exhaustion disorder: a cross sectional study**

Andreas Nelson^1,2^, Hanna Malmberg Gavelin^3,4^, Carl-Johan Boraxbekk^5,6,7^, Therese Eskilsson^8,9^, Maria Josefsson^10,11^, Lisbeth Slunga Järvholm^8,^ and Anna Stigsdotter Neely^1,12,13^

^1^Department of Social and Psychological Studies, Karlstad University, Sweden

^2^Department of Anaesthesiology, Central Hospital of Karlstad, Region Värmland, Sweden.

^3^Academic Unit for Psychiatry of Old Age, University of Melbourne, Melbourne, Australia

^4^Department of Psychology, Umeå University, Sweden

^5^Department of Radiation Sciences, Diagnostic Radiology, Umeå University, Sweden

^6^Institute of Sports Medicine Copenhagen (ISMC), Copenhagen University Hospital Bispebjerg, Copenhagen, Denmark

^7^Danish Research Centre for Magnetic Resonance, Centre for Functional and Diagnostic Imaging and Research, Copenhagen University Hospital, Amager and Hvidovre, Denmark.

^8^Department of Public Health and Clinical Medicine, Section for Sustainable Health, Umeå University, Sweden

^9^Department of Community Medicine and Rehabilitation, Physiotherapy, Umeå University, Sweden

^10^Centre for Demographic and Ageing Research (CEDAR), Umeå University, Sweden.

^11^Department of Statistics Umeå, University, Sweden.

^12^Department of Social Sciences, Technology and Arts;

^13^Department of Health, Education and Technology, Luleå University of Technology, Sweden.

**Author note**

Correspondence concerning this article should be addressed to andreas.nelson@kau.se

**Supplemental tables**

**Table S1**

*Pretest comparisons between the analysed sample of patients (n=103) and the patients excluded due to missing cognitive data (n=29)*

| Variable | Analysed | Excluded | *df* | *χ ^2^* | *t* | *p* | |
| --- | --- | --- | --- | --- | --- | --- | --- |
| Female^a^ | 88 (85.4%) | 22 (75.9%) | 1 | 1.49 |  | .22 | |
| Age^b c^ | 43.28 (8.75) | 43.62 (7.49) | 130 |  | -0.19 | .85 | |
| Range | 22-60 | 25-56 |  |  |  |  | |
| Education level^a^ |  |  | 2 | 4.10 |  | .13 | |
| Elementary school | 6 (5.8%) | 0 |  |  |  |  | |
| High school | 32 (31.1%) | 14 (48.3%) |  |  |  |  | |
| University | 65 (63.1%) | 15 (51.7%) |  |  |  |  | |
| SMBQ, total score^b c^ | 4.87 (0.96) | 4.98 (0.91)^d^ | 116 |  | -0.39 | .70 | |
| Verbal ability^b c e^ | 22.13 (4.67) | 23.60 (3.45)^f^ | 126 |  | -1.44 | .14 | |
| ^a^Based on Pearson’s Chi-square test. ^b^Mean (SD). ^c^Based on Independent Samples T-test. ^d^*n*=15. ^e^Assessed using the SRB:1, a 30-item multiple choice synonym test [^1^]. ^f^*n*=25. | | | | | | |  |

**Table S2**

*Baseline comparisons between the patients remaining in the study (n=132) and the patients who dropped out before pretest (n=29)*

| Variable | Remaining | Dropped out | *df* | *χ ^2^* | *t* | *p* | |
| --- | --- | --- | --- | --- | --- | --- | --- |
| Female^a^ | 110 (83.3%) | 22 (75.9%) | 1 | 0.90 |  | .34 | |
| Age^b c^ | 43.36 (8.46) | 41.69 (8.89) | 159 |  | 0.95 | .34 | |
| Range | 22-60 | 28-58 |  |  |  |  | |
| Education level^a^ |  |  | 2 | 0.37 |  | .83 | |
| Elementary school | 6 (4.5%) | 2 (6.9%) |  |  |  |  | |
| High school | 46 (34.8%) | 9 (31.0%) |  |  |  |  | |
| University | 80 (60.6%) | 18 62.1%) |  |  |  |  | |
| SMBQ, total score^b c^ | 5.50 (0.78) ^d^ | 5.50 (0.81)^e^ | 139 |  | 0.03 | .98 | |
| ^a^Based on Pearson’s Chi-square test. ^b^Mean (SD). ^c^Based on Independent Samples T-test. ^d^*n*=121. ^e^*n*=20. | | | | | | |  |

**Table S3**

*Correlations between the variables included in the regression analyses*

| Patients | *1* | *2* | *3* | *4* | *5* | *6* | *7* | *8* |  |  |
| --- | --- | --- | --- | --- | --- | --- | --- | --- | --- | --- |
| 1. SCCs composite | -- |  |  |  |  |  |  |  |  |  |
| 2. Global cognition composite | .13 | -- |  |  |  |  |  |  |  |  |
| 3. Exhaustion | .27^**^ | .16 | -- |  |  |  |  |  |  |  |
| 4. Depression | .30^**^ | .09 | .61^**^ | -- |  |  |  |  |  |  |
| 5. Anxiety | .25^*^ | .10 | .47^**^ | .50^**^ | -- |  |  |  |  |  |
| 6. Sex | .07 | -.02 | .12 | -.02 | -.08 | -- |  |  |  |  |
| 7. Age | -.24^*^ | -.26^**^ | .06 | -.07 | -.24^*^ | .01 | -- |  |  |  |
| 8. Education level | .12 | .23^**^ | .13 | -.06 | .04 | .08 | .07 | -- |  |  |
| Control group |  |  |  |  |  |  |  |  |  |  |
| 1. SCCs composite | -- |  |  |  |  |  |  |  |  |  |
| 2. Global cognition Composite | .01 | -- |  |  |  |  |  |  |  |  |
| 3. Exhaustion | .46^**^ | -.12 | -- |  |  |  |  |  |  |  |
| 4. Depression | .48^**^ | -.12 | .53^**^ | -- |  |  |  |  |  |  |
| 5. Anxiety | .42^**^ | -.23 | .54^**^ | .70^**^ | -- |  |  |  |  |  |
| 6. Sex | .37^**^ | -.21 | .28^*^ | .07 | .04 | -- |  |  |  |  |
| 7. Age | -.00 | -.23 | -.06 | -.12 | -.03 | .12 | -- |  |  |  |
| 8. Education level | .03 | .30^*^ | -.11 | -.24^*^ | -.13 | .13 | -.05 | -- |  |  |
| *Note.* For sex, 1=female, 0= male. For education level, 1 = university, 0 = high school or elementary school.  *^*^p < .05, ^**^p < .01, two-tailed Pearson correlations.* | | | | | | | | | | |

**Table S4**

*Partial correlations between subjective cognitive complaints and objective cognitive test results, controlling for age, sex and education level*

| Variables, patients | 1 | 2 | 3 | 4 | 5 | 6 | 7 | 8 | 9 | 10 | 11 | 12 | 13 |
| --- | --- | --- | --- | --- | --- | --- | --- | --- | --- | --- | --- | --- | --- |
| 1. PRMQ, total score | -- |  |  |  |  |  |  |  |  |  |  |  |  |
| 2. PRMQ, prospective memory | .94^***^ | -- |  |  |  |  |  |  |  |  |  |  |  |
| 3. PRMQ, retrospective memory | .94^***^ | .75^***^ | -- |  |  |  |  |  |  |  |  |  |  |
| 4. PRMQ, self-cued memory | .96^***^ | .92^***^ | .87^***^ | -- |  |  |  |  |  |  |  |  |  |
| 5. PRMQ, environmentally cued memory | .95^***^ | .86^***^ | .92^***^ | .83^***^ | -- |  |  |  |  |  |  |  |  |
| 6. PRMQ, short-term memory | .96^***^ | .91^***^ | .88^***^ | .91^***^ | .93^***^ | -- |  |  |  |  |  |  |  |
| 7. PRMQ, long-term memory | .96^***^ | .89^***^ | .91^***^ | .94^***^ | .90^***^ | .85^***^ | -- |  |  |  |  |  |  |
| 8. CFQ, total score | .77^***^ | .76^***^ | .68^***^ | .78^***^ | .70^***^ | .73^***^ | .75^***^ | -- |  |  |  |  |  |
| 9. Executive functions domain | .05 | -.02 | .11 | .07 | .03 | .04 | .06 | -.00 | -- |  |  |  |  |
| 10. Working memory domain | .09 | .13 | .04 | .07 | .09 | .08 | .08 | .01 | .42^***^ | -- |  |  |  |
| 11.Episodic memory domain | .00 | .05 | -.05 | .00 | -.00 | .01 | -.01 | -.06 | .27^**^ | .18 | -- |  |  |
| 12.Perceptual speed domain | .00 | .02 | -.02 | .04 | -.04 | -.03 | .03 | -.01 | .44^***^ | .15 | .31 | -- |  |
| 13.Reasoning ability domain | .09 | .17 | -.01 | .12 | .04 | .05 | .12 | .05 | .41^***^ | .39^***^ | .32^***^ | .42^***^ | -- |
| Variables, control group |  |  |  |  |  |  |  |  |  |  |  |  |  |
| 1. PRMQ, total score | -- |  |  |  |  |  |  |  |  |  |  |  |  |
| 2. PRMQ, prospective memory | .94^***^ | -- |  |  |  |  |  |  |  |  |  |  |  |
| 3. PRMQ, retrospective memory | .89^***^ | .68^***^ | -- |  |  |  |  |  |  |  |  |  |  |
| 4. PRMQ, self-cued memory | .95^***^ | .91^***^ | .83^***^ | -- |  |  |  |  |  |  |  |  |  |
| 5. PRMQ, environmentally cued memory | .89^***^ | .81^***^ | .83^***^ | .70^***^ | -- |  |  |  |  |  |  |  |  |
| 6. PRMQ, short-term memory | .90^***^ | .86^***^ | .78^***^ | .78^***^ | .90^***^ | -- |  |  |  |  |  |  |  |
| 7. PRMQ, long-term memory | .91^***^ | .84^***^ | .84^***^ | .93^***^ | .72^***^ | .64^***^ | -- |  |  |  |  |  |  |
| 8. CFQ, total score | .66^***^ | .63^***^ | .58^***^ | .63^***^ | .59^***^ | .56^***^ | .63^***^ | -- |  |  |  |  |  |
| 9. Executive functions domain | .03 | .07 | -.03 | .05 | -.01 | -.01 | .06 | .05 | -- |  |  |  |  |
| 10. Working memory domain | .10 | .12 | .06 | .13 | .05 | .08 | .11 | .05 | .58^***^ | -- |  |  |  |
| 11. Episodic memory domain | .17 | .26 | .02 | .12 | .21 | .16 | .15 | .22 | .37^**^ | .17 | -- |  |  |
| 12. Perceptual speed domain | -.15 | -.10 | -.19 | -.11 | -.19 | -.15 | -.12 | .00 | .44^**^ | .24 | .38^*^ | -- |  |
| 13. Reasoning ability domain | .03 | .07 | -.03 | .02 | .04 | .10 | -.05 | .19 | .59^***^ | .29^*^ | .21 | .40^**^ | -- |

*^*^p < .05, ^**^p < .01, ^***^ p < .001, two-tailed.*

**Supplemental figures**

**Figure S1**

*Flow and attrition of patients participating in the RECO trial*

**
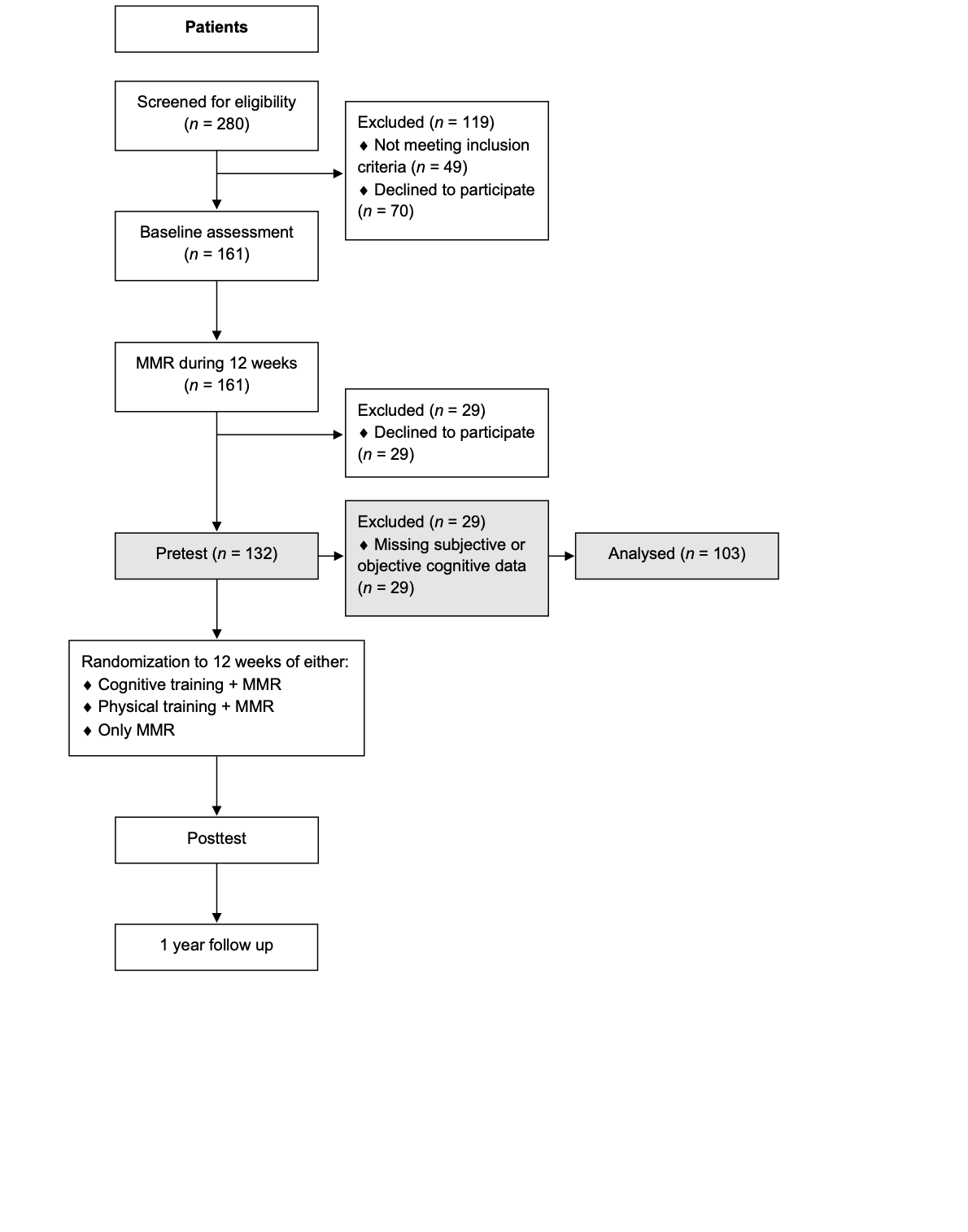
**

*Note.* Grey boxes specify the sample analysed in this study.

**References**

1. Dureman, I., Eriksson, U. B., Kebbon, L., & Österberg, E. (1971). *Manual till DS-batteriet*. Skandinaviska testförlaget.
